# A CSI-Based Indoor Fingerprinting Localization with Model Integration Approach

**DOI:** 10.3390/s19132998

**Published:** 2019-07-07

**Authors:** Yuqing Yin, Changze Song, Ming Li, Qiang Niu

**Affiliations:** 1 School of Computer Science and Technology, China University of Mining and Technology, Xuzhou 221000, China; 2 Mine Digitization Engineering Research Center of the Ministry of Education, Xuzhou 221000, China

**Keywords:** indoor fingerprinting localization, channel state information, multi-model integration

## Abstract

Convenient indoor positioning has become an urgent need due to the improvement it offers to quality of life, which inspires researchers to focus on device-free indoor location. In areas covered with Wi-Fi, people in different locations will to varying degrees have an impact on the transmission of channel state information (CSI) of Wi-Fi signals. Because space is divided into several small regions, the idea of classification is used to locate. Therefore, a novel localization algorithm is put forward in this paper based on Deep Neural Networks (DNN) and a multi-model integration strategy. The approach consists of three stages. First, the local outlier factor (LOF), the anomaly detection algorithm, is used to correct the abnormal data. Second, in the training phase, 3 DNN models are trained to classify the region fingerprints by taking advantage of the processed CSI data from 3 antennas. Third, in the testing phase, a model fusion method named group method of data handling (GMDH) is adopted to integrate 3 predicted results of multiple models and give the final position result. The test-bed experiment was conducted in an empty corridor, and final positioning accuracy reached at least 97%.

## 1. Introduction

As mobile devices get increasingly popular, the demand for positioning accuracy of indoor positioning technology accordingly becomes higher and higher. Unlike outdoor positioning that uses capitalized GPS based on line-of-sight path to pinpoint the location [1], indoor positioning is often influenced by various environmental factors, such as multipath effect [2], slow decay [3], etc. On top of that, indoor localization systems embedded in mobile devices are characterized by low complexity and short online processing time. Under this circumstance, Wi-Fi fingerprint-based indoor localization technology [4] stands out, which is sure to satisfy these requirements. A large amount of fingerprint data needs to be collected to train the fingerprint model, and this would help realize a complete real-time localization.

The implementation of fingerprint localization method is usually divided into two steps, namely the training stage and the testing stage. During the training stage, some machine-learning algorithms are adopted to train the collected data, which can reduce the complexity of calculation on positioning models. Popular machine-learning algorithms include K-Nearest Neighbor (KNN), neural networks, and support vector machines (SVM), which are universally applied in indoor localization technology [5,6]. During the testing stage, mobile devices with specific NIC (network interface card), such as the Intel Wi-Fi link (IWL) 5300 NIC [7], are used to collect data in real time. Then the trained model can predict the label of real-time data. By comparing the labels of fingerprint database, the final position of prediction results can be obtained.

Many existing indoor positioning systems usually use the receiving signal strength (RSS) data as fingerprint information [8] owing to the simplicity of the RSS of the equipment operation and the low requirements of hardware. For instance, the Horus system takes advantages of the RSS data by probability to locate [9]. However, there are two weaknesses to the method of the RSS. First, the collected RSS data will be different for the same position over time owing to the influence of multiple ways of transmission in the interior environment. Consequently, the variability of RSS data could cause large positioning errors in the same location [10]. Secondly, the RSS data could only show some vague information in some respects, which means that the impact of some fine-grained actions is not obvious on the RSS signal. Presently, some existing mobile devices with Wi-Fi NICs can be applied to obtain the channel state information (CSI), and this can obviously improve the accuracy of indoor localization [11].

In this paper, we put forward a new way of indoor positioning, which can fully exploit the characteristics of the wireless channel state information data so that the accurate detection of the object location information can be realized. Because the single model localization has a certain error rate, we use a multi-model integration strategy, which has been widely used in fields of image processing [12] and voice recognition [13]. Furthermore, the proposed method uses the wireless channel state to sense the subtle motion, which is reflected by the amplitude of the subcarrier of each antenna. Presently, most researchers are trying to use a separate algorithm model to perform localization processing on the collected CSI data. Few researchers have studied how to integrate multiple results to complete localization. This paper proposes a multi-model integration method based on group method of data handling (GHDM) algorithm. This method can determine multiple prediction results and find the optimal positioning results. Our proposed strategy used the CSI data extracted from each antenna of Intel’s IWL 5300NIC for training. Each link establishes a deep neural network model (DNN) [14], and then prediction labels are obtained by the judgment.

In particular, ModelF, a novel indoor fingerprinting method using CSI data, is proposed in this paper. First, we start with the preliminary knowledge of CSI. Then we present our system structure from the perspective of the data preprocessing, the training, and the testing stage, respectively. Local outlier factor (LOF), the anomaly detection algorithm, is used to detect and correct the abnormal CSI data. In the training phase, three antennas correspond to three data links, and each link constructs a DNN model. Then, three models sort out three predicted values. If we simply merge the three classifiers constructed, which may lead to decision redundancy and unsatisfactory localization accuracy. However, a sub-ensemble for fusion may have better results. Therefore, we propose a multi-model fusion method based on GMDH to make the fusion result better than the single classification result. During the testing phase, each model predicts the output of the test set. Then, the prediction results are analyzed and judged based on the GMDH, which contributes to the final positioning results.

The proposed ModelF method is validated with abundant experiments in the corridor. In addition, our strategy also achieves some remarkable results. The accuracy of localization could reach 97% and the mean error of localization could reach 0.0568 m. The main contributions of the paper are as follows:

1. A novel indoor localization method based on CSI fingerprinting named ModelF is proposed in this paper.
2. In ModelF, an anomaly detection method named LOF is presented to estimate outliers in CSI data. Moreover, a multi-model fusion method named GMDH is also proposed to integrate localization outputs from 3 antennas for purposes of improving localization accuracy.
3. Lots of experiments were conducted to verify the effectiveness of ModelF. Regarding the comparisons under different configurations, the localization results of ModelF showed favorable performance in localization error and running time.

The rest of the paper is organized as follows. Related works are stated in Section 2. The preliminary knowledge about CSI is presented in Section 3. The system is presented in Section 4. The experiment is discussed in Section 5. Finally, Section 6 shows the conclusion.

## 2. Related Works

With GPS unable to pinpoint indoor locations, researchers have proposed various ways to study indoor localization. Common indoor positioning methods are Wi-Fi-based, Bluetooth-based [15], RSS-based, geomagnetic [16], and so on. Among these signals, indoor localization based on Wi-Fi has received a lot of attention. Compared with other signals, Wi-Fi signals are characterized by convenience and low cost. We can do our research using a common off-the-shelf router. On the other hand, CSI information extracted from Wi-Fi is more sensitive to changes in the environment [17,18]. The CSI information is more stable and does not fluctuate significantly with time and space.

Considerable works have been researched on indoor localization methods based on CSI. In terms of the technology they used, these efforts can be categorized into 2 types: non-AI ranging method and AI-based fingerprinting method [19,20]. The first category generally extracts distance or angle information from Wi-Fi signals for localization by use of triangulation. Unlike the TOF (Time of Flight) method for RSS ranging, FILA [21] establishes the relationship equation between subcarriers value of CSI and the distances. It can reach the average latency to millisecond-level and reach the localization error to meter-level. Nevertheless, multiple Aps are necessary to make triangulation calculation possible, which may cause more cost in devices. In addition, the validity of ranging model is also affected by environmental factors. The second category requires a training phase to survey the floor plan and a test phase to search for the most matched fingerprint for location estimation. FIFS [22] and PinLoc [23] use CSI obtained through the off-the-shelf IWL 5300 NIC to build reliable fingerprints. Although these techniques need a large amount of calibration to build the fingerprint database and the latency only reaches the second level, they could achieve high localization precision to reach decimeter or centimeter level.

In addition, CSI is also used for other applications. Li et al. [17] uses Wi-Fi signals for gesture recognition. In recent years, Wi-Fi signals have gradually been used to sense activities. The researchers could “listen” to people’s words and to “see” people’s movement by the extracted wireless signal features. WiFinger was the first work to use wireless signals to sense gesture recognition. Since each gesture is unique, the impact of the gesture on the CSI value is also unique over a certain period of time. By corresponding the CSI fingerprint information and different gestures, WiFinger could recognize the meaning of the gesture by CSI.

## 3. Preliminary Knowledge

### 3.1. CSI

Channel state information can now be accessed more easily than in the past. With a specific network card, the physical layer information recorded on the hardware can be detected directly. Now, we can collect the CSI data from our laptops by accessing the device drivers. CSI can record all kinds of changes in transmission channels during transmission, and it is usually described in the form of a Channel Frequency Response (CFR) [24]. By modifying the existing network card driver, each CSI packet is a set of CFR values on various subcarriers, and the format of CSI is shown as follows.

(1) $$C=[C(f_{1}),C(f_{2}),\ldots ,C(f_{N})]$$

The Wi-Fi device currently on the market supports the IEEE802.11 standards, including the multiple transmitting (TX) and receiver (RX) antennas. Therefore, the Multiple-Input Multiple-Output (MIMO) [25] system also follows these standards. In the OFDM system [26], the channel between each pair of TX and RX comprises of multiple subcarriers and the narrow-band flat-fading channel with multiple TX-PX antennas is modeled as:

(2) Y=C×X+N

where *Y* is the received vector, *X* is the transmitted vector, *C* is the CFR value matrix and *N* is the additive white Gaussian noise. We define the number of *T* and *R* equal to the number of TX and RX antennas respectively, and M is the number of subcarriers. Then *C* is a T×R×M dimensional matrix.

The CFR value $C(f_{i})$ of subcarrier *i* is a complex value, which is defined by:

(3) $$C\left(f_{i}\right)=\left|\right|C\left(f_{i}\right)\left|\right|e^{j∠C(f_{i})}$$

$\left\|C(f_{i})\right\|$ represents the amplitude and $∠C(f_{i})$ is phase data. In this paper, our scheme mainly uses the CSI value of these 30 subcarriers to locate. According to references [27,28], for each TX-RX antenna pair, the driver of commercial Intel 5300 Wi-Fi NIC reports CSI values for 30 OFDM subcarriers of the 20 MHz Wi-Fi channel. Nevertheless, commercial software-defined radio can be used to extract all 56 subcarriers of the 20 Mhz Wi-Fi channel. What we use in the experiment was the common commercial Wi-Fi router, leading to the results that only 30 subcarriers for one TX-RX antenna pair were extracted. However, in our proposed algorithm, three antennas collect CSI signals at the same time. 30 subcarriers of each antenna can train a DNN classification model and then a fusion model algorithm is used to give the final positioning results.

### 3.2. CSI for Localization

The environment is intricate indoors. When the indoor environment changes, CSI amplitude value also can have different reactions. Because of the multipath effect and channel fading, 30 CSI subcarriers will show different amplitude values at different positions. According to the diversity of 30 CSI subcarriers at different locations, a mapping relationship can be established between location and subcarrier values.

**Figure 1:**
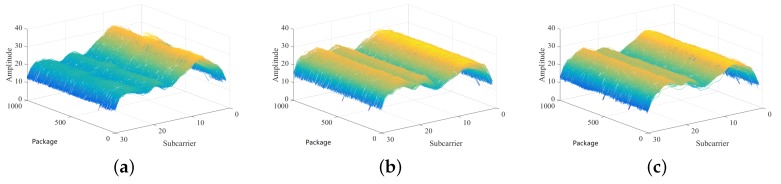
The amplitude distribution of different positions at 1000 time points. (**a**) Position 6; (**b**) Position 14; (**c**) Position 22.

Figure 1 shows the distribution of CSI amplitude values when three different points in the room are randomly assigned. We collected 1000 packets for these locations. Each packet contains the amplitude of the 30 subcarriers at this time period. The amplitude of each subcarrier is maintained at this stable value for the consecutive times. A set of CSI amplitude values in each time period maintained the same distribution. Occasionally, there are a couple of CSI amplitude values that deviate from this distribution, but we can also take a certain strategy to correct the maximum probability.

## 4. System Design

### 4.1. System Architecture

**Figure 2:**
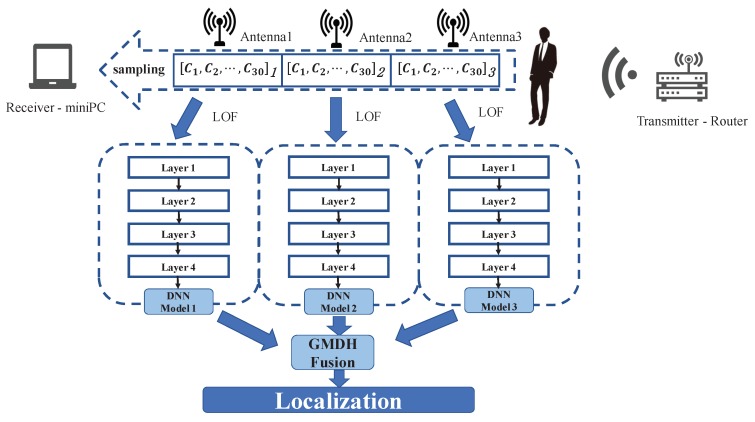
ModelF structure.

Figure 2 shows the system framework, ModelF, only needs a router and a mobile device with 5300 NIC. The mobile device can receive packets and read the CSI data. The mobile device has 3 antennas to receive CSI data. Each antenna will collect the CSI data of 30 different subcarriers. From each packet, we get 90 CSI values. When we are at a location in the wireless coverage area, it shows an impact on the transmission channel in the space. The change of the channel is described by the CSI data. Meanwhile, the receiver collects the CSI data. For different locations, the distributions of CSI data are various. Similarly, for the same point, the distribution of CSI data collected by three antennas is also different. Our system will analyze the diversity of channel changes at different points based on the CSI data collected by the three antennas. On the whole, ModelF consists of 3 phases: data preprocessing, offline training, and online testing.

In the stage of data preprocessing, ModelF uses LOF algorithm for noise reduction processing. It modifies the detected exception sample, to avoid training that affects the weight of a deep-learning network. Then, ModelF normalizes the data to eliminate the difference of data between different subcarriers. The normalization of data can improve the learning efficiency of the deep-learning network. In the offline training, ModelF is going to train 3 neural network models for each antenna to classify predicted position in the online phase and to train a GMDH network for model fusion. In contrast to most fusion algorithms, GMDH algorithm does not fuse the whole set of classification results, but continuously fuses the subsets of the set. This can effectively mine the complementary of different model outputs, thus improving the positioning accuracy of the system model. In the online testing phase, through the depth neural network and GMDH network which have been constructed, the positioning results of the real-time data collected by the system are obtained.

### 4.2. LOF Denoising

**Figure 3:**
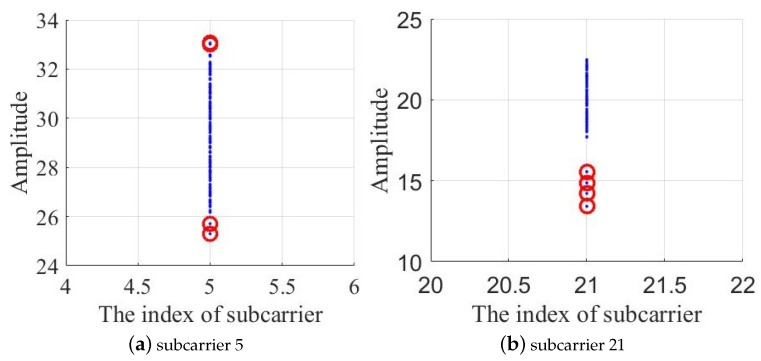
Anomaly detection based on LOF algorithm. The blue points represent the amplitude values of each subcarrier, and the point with a large degree of separation is marked with a red circle.

In the process of collecting data, some abrupt data are always collected. The abrupt change of these data may be caused by environmental changes such as the movement of non-experimenters at a certain time or equipment. ModelF uses the LOF, an anomaly detection algorithm, to find abnormal data and modify it to normal values. As shown in Figure 3, the amplitude values of the 5-subcarrier and the 21-subcarrier are plotted in the coordinate, and the point with a large degree of separation is marked with a red circle. The LOF algorithm can be used to detect and mark the points with large outliers.

The main idea of the LOF algorithm is a density-based detection method. The main idea is that given a sample data set, the degree of dispersion for the entire sample set is calculated for each sample point, i.e., the number of points within a certain distance around it is mapped to a value as an outlier factor of the sample point. If a sample point has a relatively large number of points in a certain field and the density of all points in the field is relatively high, the sample point is considered to be the normal data point, otherwise, the sample point is considered to be an abnormal data point. The density is determined by the set threshold.

The degree of dispersion of each sample point against this data set, outlier factor, is given by the following Equation (4).

(4) $$LOF_{k}\left(C\right)=\frac{\sum_{i=1}^{card(N_{k}\left(C\right))}\frac{\rho_{k}\left(P^{\left(i\right)}\right)}{\rho_{k}\left(C\right)}}{k}$$

where $\rho_{k}\left(C\right)$ is the local reachable density of the sample point *C*. $N_{k}\left(C\right)$ is the *k*th distance field of sample point *C*. *k* is the number of points in $N_{k}\left(C\right)$.

Finally, LOF randomly picks some normal points and assigns an average value of normal values to the outlier point.

### 4.3. DNN Model Training

After data processing, we need to train the model. There are two main steps: forward propagation and back propagation. The data of the training set should be normalized before the model training. The normalized method uses z-score normalization. The DNN model contains 1 input layer, 3 hidden layers, and each hidden layer has the same neuron node. $Q_{1},Q_{2},Q_{3},Q_{4}$ are defined as the number of neurons in the first, second, third, and fourth layers, and it follows that $Q_{1}=Q_{2}=Q_{3}=Q_{4}$. Both the lower and upper neurons are fully connected, which means that any neuron in the i-th layer must be connected to any neuron in the $\left(i+1\right)$-th layer. The next layer of neurons learns a linear relationship with all the neurons in the upper layer:

(5) $$z_{j}^{l}=w_{j0}^{l}x_{0}+w_{j1}^{l}x_{1}+w_{j2}^{l}x_{2}+\cdots +w_{jq}^{l}x_{q}$$

*l* is the number of layers. *j* represents the *j*-th neuron in the *l* layer. *q* is the number of neurons in the *l*-th layer. Finally, a result is output, which is the result of the predicted positioning of the model. In the DNN model, there is a complex linear relationship coefficient *w* and the bias coefficient $w_{0}$ between the upper and lower neurons. These parameters together form a matrix. Respectively, we define $W^{1}$, $W^{2}$, $W^{3}$ as the coefficient matrix of the first and second layer coefficient matrix, the second layer and the third layer, and the third layer and the fourth layer. To speed up the training, the neural network has more abundant power of expression, the output of each layer needs to pass an activation function, and we use the tanh function as the activation function [29], tanh form is as follows:

(6) $$\sigma \left(x\right)=\tanh\left(x\right)=\frac{e^{z}-e^{-z}}{e^{z}+e^{-z}}$$

When the model is trained, the forward propagation training is carried out first, and the output of each neuron is:

(7) $$a_{j}^{l}=\sigma \left(z_{j}^{l}\right)=\sigma \left(\sum_{i=0}^{q}w_{ji}^{l}x_{i}\right)$$

This method can be regarded as the problem of multi-classification. The activation function of the last layer uses the SOFTMAX function. The definition of SOFTMAX function is as follows:

(8) $$a_{j}^{l}=\frac{e^{z_{j}^{l}}}{\sum_{j=1}^{q}z_{j}^{l}}$$

A logarithmic likelihood function is used to describe the difference between the predicted results of the model and the actual label. The definition of logarithmic likelihood function is as follows:

(9) $$J(W,a^{l},y)=-\sum y_{j}\ln a_{j}^{l}$$

*y* is the sample label. Then the back-propagation algorithm [30] is carried out. The loss function can be considered to be a multivariate equation with a linear relation coefficient $w_{ji}^{l}$ as unknown. Take the partial derivative with respect to each linear relation coefficient. The value of the partial derivative is the gradient of *w* and *b* is:

(10) $$\frac{\partial J}{\partial w}=\left(a^{l}-y\right)•x^{T}$$

(11) $$\frac{\partial J}{\partial b}=\left(a^{l}-y\right)$$

The average gradient of the whole sample was taken, and the value of the linear relation coefficient $w_{ji}^{l}$ was updated with the Momentum optimization algorithm.

### 4.4. Data Fusion and Location Prediction

We use the fusion algorithm GMDH to judge the final predicted results. The basic idea is to construct a multi-layer feedforward neural network structure. First, it needs to define the mapping between input and output, and the reference function. Then it is updated iteratively through a series of operations such as heredity, evolution, variation, selection, and elimination. Finally, the optimal model is selected by the termination rule. The sample set is divided into a training set and verification set. Our input is the classification result of the three sub-classifiers $\left(r_{1},r_{2},r_{3}\right)$, and the output is the only location prediction point $f(r_{1},r_{2},r_{3})$. The relationship between input and output is expressed by the first-order K-G polynomial of three variables:

(12) $$\overset{•}{L}=f\left(R_{1},R_{2},R_{3}\right)=\theta_{1}R_{1}+\theta_{2}R_{2}+\theta_{3}R_{3}$$

$\left(R_{1},R_{2},R_{3}\right)$ represents the input matrix. ${\left(\theta_{1},\theta_{2},\theta_{3}\right)}^{T}$ is the parameter estimated by the least square method (LS) on the training set. The calculation formula is:

(13) $$\theta ={\left(R^{T}R\right)}^{-1}R^{T}Y$$

Take each sub-term in the K-G polynomial as the input of the network structure:

(14) $$x_{1}=\theta_{1}R_{1},x_{2}=\theta_{2}R_{2},x_{3}=\theta_{3}R_{3}$$

The initial input data is pair-wise combined as the input of the first layer of the network structure. we get $C_{3}^{2}$ candidate neurons of the polynomial, which is written as:

(15) $$z_{t}=\theta_{i}x_{i}+\theta_{j}x_{j}\left(i,j=1,2,3;i\neq j;t=1,2,\cdots ,C_{3}^{2}\right)$$

$\theta_{i}$ and $\theta_{j}$ are also the parameters estimated by the LS method on the training set. We define the termination rule as:

(16) $$\eta =\sum_{i=1}^{N}{\left(L-\overset{•}{L}\right)}^{2}$$

η is the overall error for all the samples. When the output of the candidate model is less than the set threshold λ, it is selected as the input of the next layer. Candidate models with outputs greater than the threshold are eliminated. λ is updated to the minimum η to participate in the next layer of determination. Repeated iteration experiments were carried out. Until the λ reaches the minimum, the optimal fusion model is found. The classification results of the sub-classifier on the verification set are substituted into the fusion model, and the final location prediction is verified.

## 5. Experiment Validation

### 5.1. Experiment Setup

**Figure 4:**
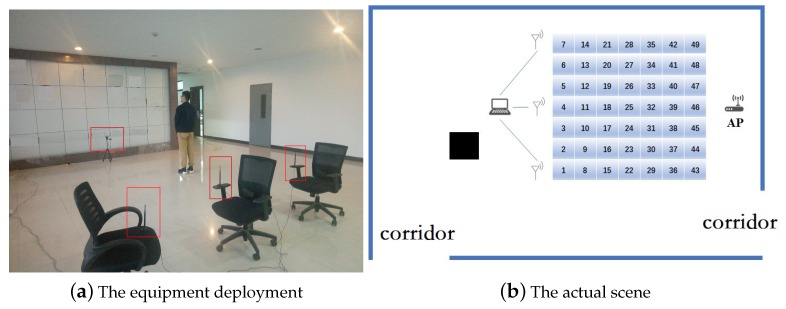
The experimental environment.

Our experiment was executed on the corner of a corridor. There were more than a dozen Wi-Fi access points in the experimental scenario. At the same time, the region was near multiple computer rooms, so the movement of indoor personnel and the electromagnetic radiation of computers could also affect the acquisition of CSI signals. Therefore, our experimental scenario was a real indoor environment and it was relatively challenging. As shown in Figure 4, the area of one square is selected and divided into 49 small squares. The experimental equipment are mainly two components: router (transmitter) and mobile device (receiver). The router is a common commercial router. The mobile device is a converted computer mainframe with a 5300 NIC. The computer is installed the 32-bit Linux system in advance, and we can change the NIC driver under Linux system installation correspondingly. The computer keeps on emitting ping command to the router and saves response packets. The response packet contains channel state information of the link. We can specify the number of CSI packets we want to collect per unit time by setting the number of Ping command. Because the CPU of the computer mainframe operation is fast and accurate, we can set the sensitivity to simulate the Wi-Fi coverage area size, and can avoid some unexpected actions by setting the sensitivity of the influence of the experimental data acquisition. With the Linux operating system, we can directly control the network card to open and close the receiving function. The CSI data can be saved directly by accessing the text file in the Linux system.

### 5.2. Experimental Difficulties Analysis

Our experiments are divided training phase and testing phase. During the training phase, a large number of CSI data collected at different locations were used to train the model. After training, a suitable linear relationship coefficient is obtained between layers of the model. During the testing phase, we need to input the CSI data into model in real time. Through the method of model fusion, multiple prediction results are analyzed and compared, and the results are obtained. In the experiment, our biggest challenge was the acquisition of data and the fusion of final results. CSI is very sensitive to changes in location. When collecting data in each small grid area, the data we collect must be suitable and abundant. Only reasonable data can fully summarize the possible distribution of CSI data in this area. If not much data is collected in the community, positioning accuracy will not be guaranteed in the prediction phase. If the collected data in the community are very dense and large, it would be very time-consuming in the training phase. It is possible to do a lot of repetitive work in the experiment, which increases the difficulty of the experiment. In the experiment, the size of our small area was 0.5 m × 0.5 m. In this area, we randomly stood sampling 5 times and collected 100 packets at a time. Finally, the training data for each area has 500 CSI packets. The data of the 5th sampling is used to test system. Finally, the prediction results of the three models needs to be fused based on the GHDM method.

For each location, there will be a prediction label and a reference label. The position coordinates of the predicted results are (x,y), and the actual position coordinates are $\left(\overset{\wedge }{x},\overset{\wedge }{y}\right)$. For each sample, by comparing the prediction label and the reference label, we can calculate the predicted distance error of the sample. in the form of:

(17) $$\varepsilon =\sqrt{{\left(x-\overset{\wedge }{x}\right)}^{2}+{\left(y-\overset{\wedge }{y}\right)}^{2}}$$

Table 1 listed the maximum error, minimum error, and average error of our system for all test data sets. The maximum distance between the predicted label and the actual label is the maximum error in the sample collected. The minimum distance between the predicted label and the actual label is the minimum error. In the sample collected, the average distance of the predict label and actual label is average error for all test sample. Furthermore, it was also recorded that the testing time for one DNN model was 0.006 s and the time of multi-model fusion was 0.055 s, thus the total of real-time testing time could be 0.073 s, which is considered to meet the real-time requirement.

### 5.3. Data Collection and Denoising

Our experiment requires at least two participants. A member, as an operator, is responsible for saving and collecting CSI data on the terminal. Another member, as a practitioner, moves between different positions in the experimental area. In the experiment, data were collected in 49 small regions, and each small area was sampled 5 times. The practitioner enters the zone 5 times, and randomly stands in the region. In the sample data set, the data of the first 4 samples were used as the training points, and the last sample was used as the test points. Therefore, these 49 small regions are both reference and prediction positions. At each sampling, 100 packets were collected as samples. The collection of CSI data is completed by the receiving device. The transmitter had a transmitting antenna. In addition, the receiver had three receiving antennas. There are three data collection links, and each link collects 30-subcarrier amplitude values. The collected data are processed by denoising, and then entered into the model for training to predict the predicted data.

**Figure 5:**
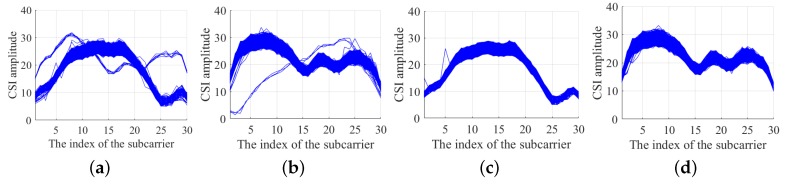
CSI data processing. (**a**,**b**) are original data; (**c**,**d**) are processed data.

We collected the 30 CSI amplitudes from 1 antenna for a period of time. Figure 5a,b display the raw CSI amplitude value collected from the receiver. The *x*-axis represents the index of the subcarrier. The *y*-axis represents the CSI amplitude value. It can be observed that some isolated curves do not conform to the trend of most curves. Through a large number of experiments, respectively, we find that the amplitude of the subcarriers has the same distribution. In other words, in a relatively short period of time, the CSI amplitude value should fluctuate within a certain range. Using this feature, we can set a maximum and lower limit according to the collected CSI amplitude value. Then an LOF algorithm is applied to detect the outliers [31]. Three LOF algorithms are used for three links. As shown in Figure 5c,d. It is shown that the abnormal points in the CSI data are corrected effectively after the LOF algorithm is modified.

### 5.4. System Performance

This section is going to show the comparison results between ModelF and the approaches that replace variables, such as CSI with RSS, DNN with SVM, and GMDH with other optimization methods.

#### 5.4.1. RSS and CSI

**Figure 6:**
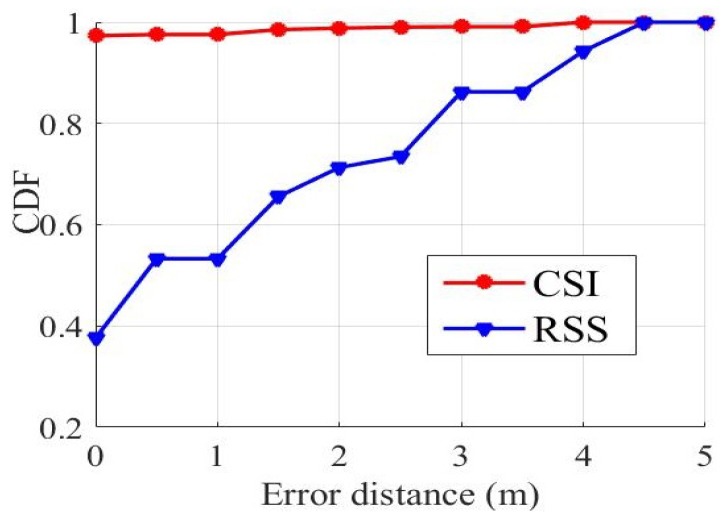
Localization precision of different sources of data.

ModelF takes CSI data as fingerprints, while many researchers take RSS data as fingerprints. This subsection is going to compare the 2 different types of fingerprints in ModelF. Our experimental devices can collect both CSI data and RSS data at the same time. As for CSI data, ModelF uses LOF denoising method to remove the abrupt data or outliers. As for RSS data, their values are thought to be inversely proportional to distances, and RSS is less sensitive to environmental changes than CSI, so there are few outliers in RSS data. However, RSS signals were also processed by a smooth filter. Table 2 shows the predict result of the maximum error, the minimum error, the average error that are calculated using different data when collecting CSI and RSS data separately. The average error of CSI is very low. The maximum distance error based on RSS is 4.4721 m, while CSI is only 3.8079 m. Figure 6 shows the cumulative distribution functions (CDF) of distance errors calculated by using RSS and CSI data for localization. For discrete variables, CDF value is the sum of probabilities for all values less than or equal to *x*, it can be represented by Equation (18).

(18) CDF(x)=P(X≤x)

In other words, CDF can express how many proportions of the predicted locations are within the error distance *x*. As shown in Figure 6, the red curve is the result of using CSI data test and the blue curve is the result of using RSS data test. When the distance error is 0, it means that the prediction label for the sample is the same as the actual label. In other words, according to the eigenvectors of the sample, the sample is directly located in the correct position. By use of RSS values, about 40% of the samples were classified to the correct positions. While by use of CSI values, about 97% of the samples were correctly positioned. From other perspective, the positioning errors of all predicted positions are within 4 m by use of CSI values, while the positioning errors of all predicted positions are within 4.5 m by use of RSS values, which reflects that CSI is more suitable for fingerprint localization.

#### 5.4.2. SVM and DNN

**Figure 7:**
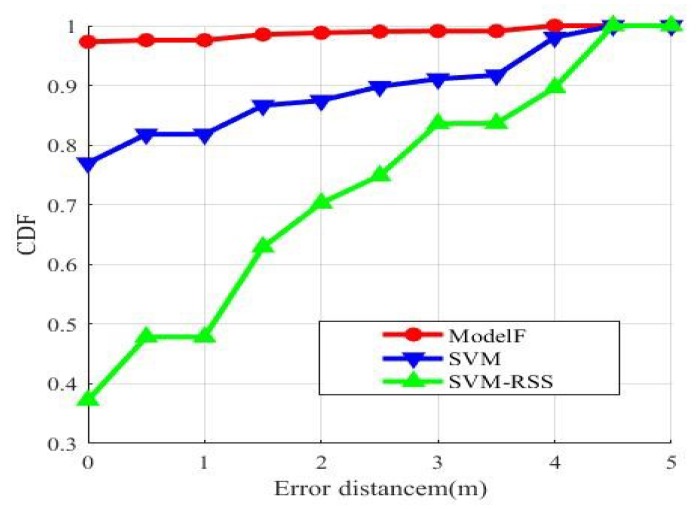
Localization precision of different classifier.

The classification model in our method uses a deep-training network. Support vector machine is also a very good classification model. Zhou et proposed an indoor positioning method SVM based on support vector machine. They used principal component analysis to extract data and reduce dimension. Then the support vector machine is used to deal with the data. The biggest difference between our method and Zhou’s is the difference of classifier. Support vector machine is a very good binary classifier, but it always takes a lot of time to deal with multiple classification problems. In addition, If the dimension of the sample eigenvector is too small, the prediction result of SVM will be very poor. We train the collected data in different classification models, which is the deep-training network and support vector machine. Table 3 shows the predict result of the maximum error, the maximum error, the average error that are calculated using different classification model. The average error is similar. The maximum distance error based on DNN is 3.8079 m, while SVM is only 4.4621 m. Figure 7 shows the cumulative distribution functions of distance errors after training with these two classification models.

The red curve is the result of test with DNN classification model. The blue curve is the test result of SVM classification model. With DNN positioning, about 97% of the samples were correctly positioned, and about 99% of the test points have an error under 3 m. With SVM positioning, only about 76% of the samples were correctly located, and about 90% of the test points have an error under 3 m. The distance error of using DNN classification model is within 4 m. The distance error of using SVM classification model is within 4.5 m. The green curve is the result of the collected RSS sample using an SVM. The correct localization of the sample is about 40%. With the same classifier, CSI performs better than RSS. Obviously, DNN has a better performance.

#### 5.4.3. Model Integration and No Integration

**Figure 8:**
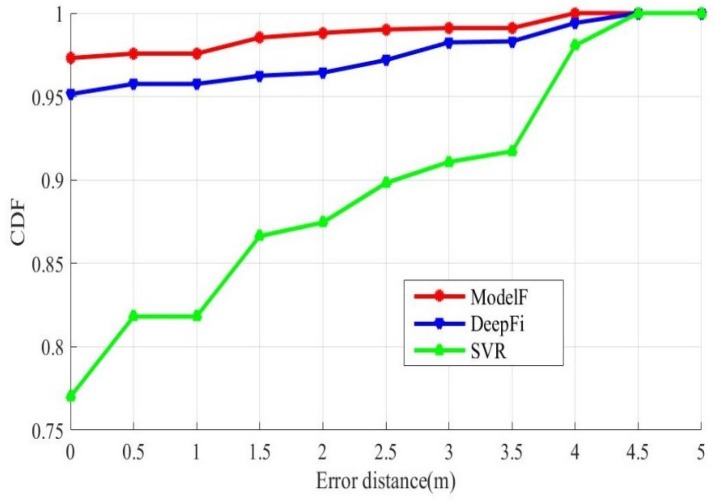
Localization precision of different method.

Our method trains and predicts the data on three links, respectively. Then the three results are judged according to the GHDM, and the only result is calculated. Our method ensures the diversity of CSI data collected by each antenna. Wang et al. proposed an indoor positioning method based on fingerprint, which called DeepFi. DeepFi used restricted Boltzmann machine algorithm (RBM) to reconstruct CSI data collected. Then the Bayes’ law is used to analyze the location. The biggest difference between our method and DeepFi’s is that we use the GMDH to determine the prediction results of multiple models. Table 4 shows the average error, maximum error, and minimum error of all samples under different input sample dimensions. Figure 8 shows the cumulative distribution functions of distance errors.

The red curve adopts the prediction result of the integration model. The blue curve is the result of direct prediction without integration. With the integration model method, about 97% of the samples were correctly positioned, and about 99% of the test points have an error under 3 m. Without integration method, only about 95% of the samples were correctly located, and about 98% of the test points have an error under 3 m. The distance error of using the integration model method is within 4 m. The distance error of without integration method is within 4.5 m. The CSI data collected by the three antennas are separately classified by SVM. The green curve is the CDF curve after integrating the three prediction results. The correct localization of the sample is about 75%. The integration model DNN has a better performance.

### 5.5. The Influence of System Parameters

#### 5.5.1. Impact of LOF

**Figure 9:**
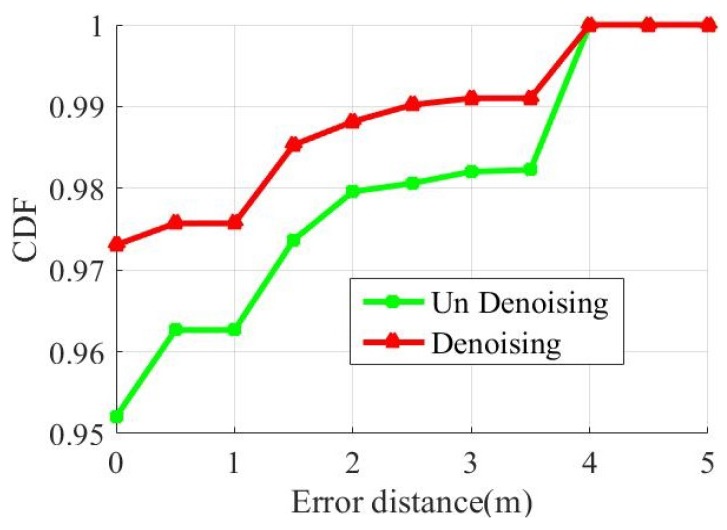
Localization precision with denoising and no-denoising.

To evaluate the effect of abnormal data on our positioning performance, we conducted two experiments. In the first experiment, we trained the CSI directly. In the second experiment, we used LOF algorithm to perform abnormal detection. Then the processed data is trained. Figure 9 shows the cumulative distribution functions of distance errors that uses detected and undetected to locate.

The red curve is the predicted result after denoising. The green curve is the prediction result of before denoising. About 95% of the samples were correctly positioned before denoising, and 97% were correctly positioned after denoising. Both have an error under 4 m for all sample. After noise reduction, the accuracy of the ModelF is improved.

#### 5.5.2. Impact of Different Antennas

In this section, some experiments have done to verity that the performance of the fusion of three antennas is better than that of the independent work of each antenna from the aspect of ROC (Receiver Operating Characteristic) curves and CDF curves.

First, it is necessary to define true positive (TP), false negative (FN), false positive (FP) and true negative (TN). If an instance is positive and predicted to be positive, it belongs to TP, while if an instance is positive and predicted to be negative, it belongs to FN. If an instance is negative and predicted to be positive, it belongs to FP, while if an instance is negative and predicted to be negative, it belongs to TN. Then, the TPR (true positive rate) can be calculated by TP/(TP + FN) and the FPR (false positive rate) can be calculated by FP/(FP + TN), which make up the *y*-axis and *x*-axis of ROC curve, respectively. For a two-category, the prediction results are sorted according to the probability from high to low, and each probability is used as a threshold for judging positive and negative, and finally the TPR and FPR values are calculated and plotted. According to the prediction results of independent work of each antenna and the fusion of three antennas, we have drawn the 4 ROC curves as follows.

**Figure 10:**
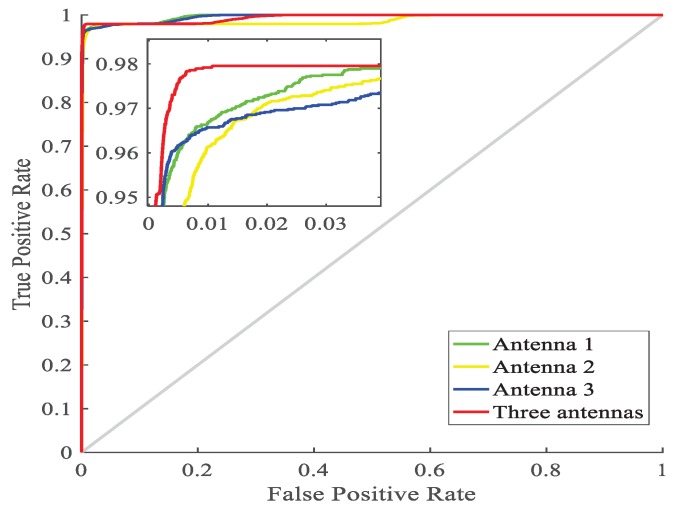
ROC curves under different antennas.

As shown in Figure 10, 4 curves near coordinate (0,1) are enlarge for better illustration. The point (0,1) is the ideal target, i.e., the closer the ROC curve is (0,1), the better the predicted results are. It can be shown in the magnified view, the prediction result of the fusion of 3 antennas is better than that of independent work of each antenna duo to the fact that the red curve is closer to point (0,1) than other 3 curves.

**Figure 11:**
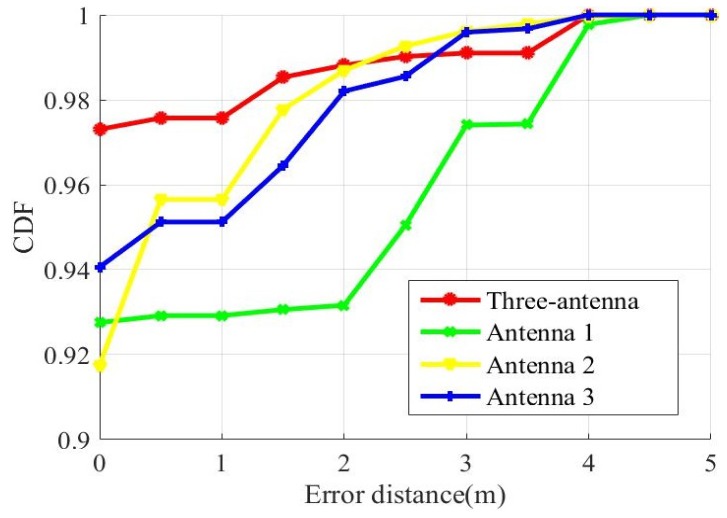
Localization precision with different antenna.

Figure 11 shows the cumulative distribution functions of distance errors that use a single antenna to locate and use three antennas to locate. In addition, we set other parameters of the experiment to remain the same.

We compared the three-fusion method with the single-antenna method. According to the CDF of estimation errors, more than 97% of the test sample have an error under 1 m with fusion method. In addition, have only 95% an error under 1 m with single-antenna method for the antenna-one and antenna-two. The third antenna has only a 95% of sample that have an error under 2.5 m. The three-fusion method better localization accuracy than the single-antenna method. Because we fuse the advantages of the three antennas.

#### 5.5.3. Impact of Different Activation Function

**Figure 12:**
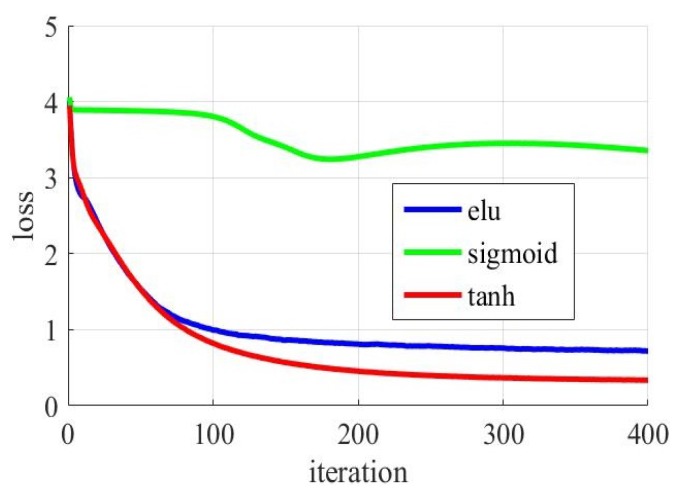
Localization precision with different activation function.

In the deep-training network model, the activation function is in various forms. To study the effect of different activation functions on our experimental results, different activation functions were used to experiment. Common activation functions are “sigmoid” function, “tanh” function and “elu” function. Change the activation function in the neural network model for the first antenna. The three activation functions are used in three experiments. Record the change of loss value during model training. We set other parameters of the experiment to remain the same. As shown in Figure 12. The *x*-axis represents the number of iterations, and the *y*-axis represents the gap between the predicted label and the actual label during the gradient descent. As the number of iterations increases.

The function of convergence is very bad for the classification model of the sigmoid. Its loss is almost undiminished. However, the loss value of the function of convergence was significantly reduced for the classification model of the tanh and the elu. Loss of the tanh model decreases faster. This indicates that the model training of the tanh model is faster and more effective.

#### 5.5.4. Impact of Different Optimization Method

In the deep-training network model, the linear relation coefficient of the neuron is updated with gradient descent method. The simplest gradient descent method may cause the loss function in model training to be less effective due to the unreasonable initial value of the update parameter. To overcome this problem, the researchers propose several optimization methods. The optimization method in our system adopts the Momentum’s optimization algorithm. At the same time, the Adam’s optimization algorithm and the RMSprop’s optimization algorithm are used to experiment. In the three experiments, the loss value of model training was changed under different optimization methods. We also set other parameters of the experiment to remain the same.

**Figure 13:**
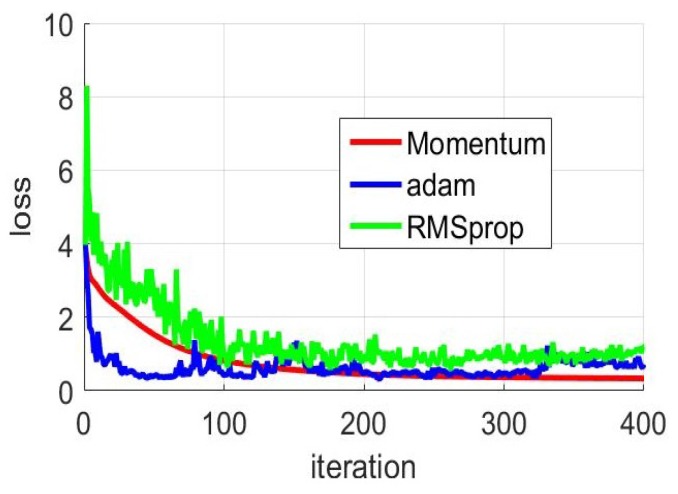
Localization precision with different optimization method.

As shown in Figure 13 The learning efficiency parameters of the three optimization methods are all 0.01. With the increase of the number of iterations, all three methods have the effect of accelerating the convergence of functions. The loss values of the three methods are all decreased from the beginning to the later stage. It is obvious that the Momentum’ optimization algorithm has the best performance. The Momentum’ optimization algorithm is very stable in the process of convergence, and the loss value continues to decrease until the function value converges. In comparison, the loss value fluctuates greatly in the convergence of functions. Especially based on Adam’s optimization method, the loss value jump is obvious. As can be seen from the three curves, neither Adam’s nor RMSprop’s method found the optimal situation. At the 400-th iteration, the loss value of the Momentum’ optimization algorithm was approximately 0.3, while the loss value of the other two methods was around 1.

## 6. Conclusions

This paper has researched indoor localization scheme-based channel state information. Due to the development and support of OFDM and MIMO technology, our method can be realized. When data are collected at different locations, the amplitude distributions of the 30 CSI subcarriers are different. According to this feature, we propose the ModelF scheme. First, CSI data are collected through three antennas and denoised by LOF algorithm. Then, three DNN models are established to train the pre-proposed data. After the model training, a certain number of samples are extracted from the training samples to evaluate the model. Next, in the testing stage, the three models will give their respective prediction results for real-time CSI data. According to the prediction results given by three models, a multi-model fusion strategy named GMDH is put forward to give the final localization result. A test-best experiment has been conducted to establish the effectiveness of ModelF from the perspective of localization accuracy and real-time requirement, where the mean localization error was 0.0568 m, and the testing for real-time data was 0.073 s.

## Figures and Tables

**Table 1:** The maximum, minimum, and mean of distance error value of ModelF.

	Max Error	Min Error	Mean Error
ModelF	3.8079	0	0.0568

**Table 2:** The maximum, minimum, and mean of distance error value of the CSI and RSS.

	Max Error	Min Error	Mean Error
CSI	3.8079	0	0.0568
RSS	4.4721	0	1.3047

**Table 3:** The maximum, minimum, and mean of distance error value of the DNN and SVM.

	Max Error	Min Error	Mean Error
DNN	3.8079	0	0.0568
SVM	4.4621	0	0.5326

**Table 4:** The maximum, minimum and mean of distance error value of the integration and no integration.

	Max Error	Min Error	Mean Error
Fusion	3.8079	0	0.0568
No Fusion	4.3012	0	0.1261
